# Interpretable Machine Learning to Anticipate the Diagnostic Yield of EEG in the Emergency department. The EMINENCE study

**DOI:** 10.1007/s10916-026-02397-y

**Published:** 2026-05-01

**Authors:** Maenia Scarpino, Ester Marra, Piergiuseppe Liuzzi, Benedetta Piccardi, Peiman Nazerian, Ilaria Sgrilli, Andrea Mannini, Andrea Nencioni, Antonello Grippo

**Affiliations:** 1 Neurophysiopathology Unit, Careggi University Hospital, Florence, Italy; 2 IRCCS Fondazione Don Carlo Gnocchi ETS, Via di Scandicci 269, Florence, Italy; 3 Stroke Unit, Careggi University Hospital, Florence, Italy; 4 Emergency Department, Careggi University Hospital, Florence, Italy

**Keywords:** Emergent EEG, Emergency department, Machine-Learning, Clinical decision support systems.

## Abstract

**Introduction:**

Emergent electroencephalography (emEEG) is increasingly employed in the emergency department (ED) for evaluating altered consciousness and seizure-related conditions, yet standardized criteria guiding its use remain limited.

**Methods:**

We retrospectively analyzed 1,018 patients (mean age 66 ± 20 years; 48.4% female) undergoing emEEG at the ED of the Careggi Teaching Hospital (Florence, Italy) in 2023. Clinical, anamnestic, and neuroimaging data available at admission were used to train supervised machine-learning (ML) models. We evaluated tree-based ensembles (Random Forest and XGBoost) to predict abnormal and epileptiform emEEG, as well as confirmation or refutation of initial diagnosis. Ground-truth labels were derived from a multidisciplinary expert team including neurologists, neurophysiopathologists and intensivists. Model performance was assessed with 5 × 5 nested cross-validation, receiver operating characteristic (ROC) analysis, balanced accuracy, decision-curve analysis, and Shapley Additive Explanations (SHAP) interpretability.

**Results:**

Abnormal emEEG occurred in 691 cases (67.9%), epileptiform activity in 192 patients (18.9%). emEEG ruled out the initial diagnostic suspicion in 514 cases (50.5%) and confirmed it in 188 cases (18.5%). Best performance was obtained with Random Forest for abnormal emEEG (AUC 0.79, 95% CI: 0.76–0.82) and diagnosis rule-out (0.84, 0.81–0.86), and with XGBoost for epileptiform emEEG (0.82, 0.78–0.85) and diagnosis confirmation (0.82, 0.79–0.85). Performance varied by initial diagnostic suspicion, but subgroup-stratified analyses showed overall consistent patterns. Key predictive features included altered consciousness, prior brain lesions, antiseizure therapy, and seizure-related presentations. Interpretability analyses revealed seizure-centric features drove confirmation, while systemic or nonspecific features favored refutation.

**Conclusions:**

Interpretable ML models using only admission data can predict emEEG outcomes and anticipate their diagnostic contribution, supporting triage and decision-making in emergency neurology without replacing clinical judgment. Models and explanations were easily usable on a freely-accessible website (www.emergencyeeg.com), where tools return probabilistic outputs for all four prediction tasks together with per-patient explanation plots, enabling transparent and reproducible use.

**Clinical Trial Number:**

Not applicable.

**Supplementary Information:**

The online version contains supplementary material available at 10.1007/s10916-026-02397-y.

## Introduction

Emergent EEG (emEEG) is increasingly employed in emergency department (ED) clinical practice for the detection of a wide range of neurological conditions, particularly in the evaluation of patients presenting with altered states of consciousness, a critical condition requiring prompt diagnosis and appropriate therapeutic management [1–6]. Early consensus efforts and subsequent policy statements have emphasized the importance of EEG in urgent pathways, especially for seizure-related presentations and status epilepticus, while also highlighting heterogeneity in ED implementation [7–10]. The rapid availability of emEEG, a reliable, non-invasive bedside tool, together with its complementary role to neuroimaging, is central to its clinical value. Unlike computed tomography or magnetic resonance imaging, which primarily provide structural information, emEEG offers functional insights into brain activity. It can detect disorders such as non-convulsive status epilepticus (NCSE) or metabolic and septic encephalopathy, which are often invisible to neuroimaging yet strongly associated with impaired consciousness [11–13]. These features explain the growing demand for emEEG in the ED setting.

Nevertheless, the diffusion of emEEG in routine emergency practice has largely depended on institutional performance capacities and the individual expertise of treating physicians, rather than standardized diagnostic criteria or universally accepted indications. Although frameworks exist for EEG terminology and seizure/status guidance, actionable recommendations in the ED remain uneven and have historically focused on NCSE and seizure presentations [8–10]. For neurological conditions other than NCSE, available data are comparatively scarce, heterogeneous in design, and often restricted to small samples, limiting generalizability and robust comparison across studies [1, 2, 4, 14–17]. Recent ED series have begun to characterize seizure detection and the clinical impact of EEG in contemporary workflows, but findings remain variable across settings [17].

In one of the few larger investigations in this setting, the EMINENCE study systematically analyzed emEEG findings in a substantial cohort of ED patients with diverse neurological disorders, demonstrating correlations between presenting diagnoses, neurological symptoms at admission, and emEEG abnormalities, while also identifying predisposing factors for pathological recordings [6, 18]. That work underscored the ED as a unique clinical environment in which physicians are frequently compelled to make rapid diagnostic and therapeutic decisions, often without immediate access to advanced diagnostics such as brain MRI or on-site specialist consultation. In this context, access to emEEG may require prioritization for time-sensitive decisions. Rapid-EEG systems are a pragmatic option to accelerate ED acquisition [19, 20], as they allow quick bedside recordings with minimal training; however, feasibility still depends on device availability and requires timely expert interpretation. Complementing rapid acquisition, prior EEG-ML work has explored signal-based automated analysis to potentially shorten interpretation time once a recording is available [21, 22]; however, this still presupposes that an emEEG has been obtained.

Building on these considerations, the present study integrates clinical-anamnestic data, neuroimaging findings, and initial ED diagnoses in supervised machine-learning models to support early emEEG prioritization. In particular, it addresses the practical need to identify patients more likely to require immediate emEEG versus those for whom acquisition may be scheduled later when immediate testing is not feasible for all patients. The primary objective was to predict emEEG outcomes, distinguishing normal from abnormal recordings and classifying epileptiform versus non-epileptiform patterns. A secondary aim was to evaluate the contribution of emEEG to diagnostic reasoning by predicting whether emEEG would confirm or refute the initial diagnostic suspicion formulated at ED admission. Beyond predictive performance, we emphasize interpretability: transparent models can clarify which clinical and anamnestic features most strongly influence predictions, thereby supporting clinician decision-making and fostering trust in decision-support tools [23–25]. To facilitate external use, reproducibility, and transparent interpretation beyond our center, we deployed the models on a publicly accessible institutional web platform that generates predictions and SHAP visualizations from user-entered admission data without storing any inputs.

## Methods

### Study design and patient selection

The EMINENCE study is an observational, single-center, retrospective study conducted at the tertiary Careggi Teaching Hospital, Florence, Italy. All patients ≥ 18 years admitted to the ED between January 1, 2023 and December 31, 2023 who underwent an emEEG were consecutively enrolled. Clinical, anamnestic, and neuroimaging data were obtained from medical records, together with disposition (discharge or hospitalization). We included emEEGs performed both during and outside normal hospital hours.

### EEG recording and classification

EEG was available during normal hours (08:00–19:00 Monday-Friday; 08:00–14:00 Saturday) and on a 24-h on-call basis outside those times for emergencies related to status epilepticus detection. Standard 30-minute EEGs were acquired with a digital system and pre-wired cap using 19 electrodes positioned according to the international 10–20 system (Fp1, Fp2, F7, F8, F3, F4, C3, C4, T3, T4, P3, P4, T5, T6, O1, O2, Fz, Cz, Pz) at a sampling rate of 128 Hz. During review, digital filters (low-pass 30–70 Hz; time constant 0.1–0.3 s; 50 Hz notch) and sensitivity (2–10 µV/mm, standard 7 µV/mm) were adjusted as needed. Studies were usually performed without sedation. EEGs were interpreted by expert neurologists/neurophysiologists, with priority given to ED requests. Recordings were classified using the American Clinical Neurophysiology Society standardized terminology [9]. Descriptors included continuity, voltage, reactivity and spontaneous variability, anterior-posterior organization, symmetry, frequency, epileptiform discharges and slow waves, periodic and triphasic patterns, sleep transients, and the presence of electrographic seizures or status epilepticus.

### Data collection protocol

Data were retrieved retrospectively from the electronic health records (EHR) of all ED patients undergoing emEEG. Predictor variables were extracted from information available at (or before) the time of emEEG request, including triage documentation, the ED physician assessment note, prehospital reports when available, and radiology reports for neuroimaging performed prior to EEG. Trained investigators (M.S., I.S.) performed structured chart abstraction and disagreements were resolved by consensus and, when necessary, adjudicated with a senior clinician (A.G.). Variables were coded as documented in routine ED care; we did not retrospectively re-adjudicate symptom semantics, in order to preserve real-world ED conditions and personnel seniority.

Demographics included age (years, continuous) and sex (binary). Initial diagnostic suspicion refers to the working diagnostic impression recorded by the treating ED physician at the time of emEEG ordering (before EEG results). It was coded as present/absent for the following categories: transient loss of consciousness (LoC), altered consciousness, stroke, transient ischemic attack, epileptic seizure, absence-like episode, status epilepticus, transient global amnesia, head trauma, fall of unknown dynamics, no specific suspicion, and “other” (rare categories grouped).

Signs and symptoms at presentation were coded as present/absent based on ED physician documentation. They included confusion, speech disorder, amnesia, headache, weakness, cranial nerve deficits, sensory symptoms, visual symptoms, hallucinations, motor manifestations, morsus (tongue bite), and transient loss of consciousness; we also recorded whether symptoms had regressed before EEG acquisition (i.e., partial or complete clinical resolution documented before the recording). Operational definitions were as follows: morsus was coded when tongue bite was explicitly documented; weakness indicated a focal or generalized motor deficit documented on presentation (e.g., hemiparesis/monoparesis or clearly described motor weakness); absence-like episode indicated a brief episode of impaired awareness/staring or unresponsiveness without collapse and without prominent convulsive activity, when documented as such; altered consciousness indicated reduced arousal or fluctuating mental status not meeting complete LoC (e.g., somnolence, stupor, marked drowsiness, or reduced responsiveness) as recorded in the chart; LoC indicated a transient episode described as loss of consciousness/unresponsiveness; confusion indicated disorientation or impaired attention/awareness documented by staff (e.g., “confused”, “disoriented”, “mental status altered” without explicit reduced arousal).

Anamnestic variables were abstracted primarily from the ED physician history section and witness descriptions recorded in the chart, supplemented by prehospital documentation when available. We coded whether the critical episode was witnessed by healthcare professionals (HCPs) (i.e., directly observed and documented by EMS personnel or ED staff) or witnessed by others (family members, caregivers, or bystanders as recorded). Past medical history variables included prior neurosurgery, known epilepsy, and known brain lesions. Known idiopathic epilepsy was coded when a prior epilepsy diagnosis was documented without an identified structural/metabolic cause (idiopathic/genetic or unspecified non-lesional epilepsy, per recorded history). Secondary epilepsy was coded when epilepsy was documented as symptomatic/structural (e.g., post-stroke, post-traumatic, tumor-related, or otherwise associated with a known lesion). Brain lesions were coded when a known structural brain lesion was documented in the history and/or prior records available in the EHR.

Acute medical conditions documented at admission were coded as present/absent and included fever, sepsis, metabolic derangements, electrolyte disturbances, and intoxication. Medication history captured antiepileptic drug therapy at admission. Comorbidities included hypertension, diabetes, dyslipidemia, thyroid disorders, and cardiac conditions.

ED neurological conditions captured neurological conditions documented as known or suspected at ED admission, including pre-existing neurological diagnoses and/or acute neurological pathologies identified on neuroimaging performed before EEG. These were coded as present/absent for subdural hematoma, brain tumor, neurodegenerative disease, head trauma, intracerebral hemorrhage, subarachnoid hemorrhage, cerebral ischemia, multi-infarct disease, and other conditions (grouped). Neurology referral was coded when a formal neurology consultation request was placed during the ED stay prior to disposition (yes/no); this reflects local workflow in which emEEG can be ordered directly by ED physicians and neurology consultation is requested selectively. Demographic data were entered into the EHR by nursing staff as part of the routine ED triage; all other variables were documented in the EHR by the ED physicians on duty during the index visit, in line with standard Italian ED triage procedures and clinical documentation guidelines.

EEG outcomes were coded as four binary targets: abnormal emEEG, presence of epileptiform activity, and whether emEEG confirmed or ruled out the initial diagnostic suspicion (definitions and labelling procedure are provided in the Outcome measures section). All categorical predictors were coded as 0/1; age was retained as a continuous variable.

### Outcome measures

Four binary outcomes were defined based on qualitative emEEG assessment. Abnormal emEEG was defined as the presence of any abnormal finding on qualitative emEEG interpretation, whereas normal emEEG indicated the absence of abnormal findings (normal, *n* = 327; abnormal, *n* = 691). Epileptiform emEEG was defined as the presence of epileptiform activity, whereas non-epileptiform emEEG indicated its absence (epileptiform, *n* = 192; not epileptiform, *n* = 826). Abnormal and epileptiform labels were assigned by two independent neurophysiologists (A.G., M.S.), and agreement between the two evaluations was required for the final label. Two additional outcomes captured the relationship between emEEG findings and the admission diagnostic suspicion formulated before EEG acquisition. emEEG confirming the initial diagnosis was defined when the admission suspicion remained the final diagnostic conclusion and emEEG provided the key supportive evidence (confirming, *n* = 188; not confirming, *n* = 830). emEEG ruling out the initial diagnosis was defined when the final diagnostic conclusion differed from the admission suspicion and the change was specifically attributable to emEEG findings (ruling out, *n* = 514; not ruling out, *n* = 504). Confirming and ruling out labels were defined by a multidisciplinary expert team (neurologists, neurophysiologists, and intensivists) with access to all clinical information available up to that time point (including presenting features, history, available investigations/imaging, and the emEEG report).

### Statistical and Machine-Learning Analysis

Medians and interquartile ranges (IQRs) were reported for continuous variables, while absolute/relative frequencies were used for binaries. Four supervised classifiers were developed, one for each outcome: abnormal emEEG, epileptiform emEEG, emEEG ruling out the initial diagnosis, and emEEG confirming the initial diagnosis. Models were trained using 58 predictors at ED admission, retrieved from ED records and detailed in the previous section. We considered Random Forest and Extreme Gradient Boosting (XGBoost), using a 5 × 5 nested cross-validation strategy. The dataset was randomly partitioned at the participant level into five outer folds; in each iteration, one fold (20%) served as the test set and the remaining folds (80%) as the training set, so that each participant appeared in the test set exactly once across outer folds. This design prevented any subject overlap between training, validation, and test subsets within each iteration. Results were aggregated across test folds. On each training set, an inner 5-fold loop performed hyper-parameter optimization, with the explored parameter ranges reported in Supplementary Table S1. The best-performing algorithm on validation was carried forward to test evaluation. Performance was assessed by balanced accuracy, Receiver-Operating Characteristic (ROC) analysis, and decision-curve analysis (DCA). To quantify uncertainty around discrimination, we computed the area under the ROC curve (AUC) with 95% confidence intervals (CIs). AUC and its variance were first estimated with the nonparametric DeLong method, and 95% CIs were reported. We also performed precision-recall analysis and reported the F1-score and average precision (AP). All estimates were obtained from test predictions by concatenating, for each outcome, the true test labels and the corresponding predicted class probabilities from the outer cross-validation folds (i.e., no model was refit for these analyses). Furthermore, we evaluated model performance within clinically meaningful subgroups defined by the binary indicators capturing the initial diagnostic suspicion at ED admission. For each outcome and diagnostic subgroup, we computed: the number of patients, the observed event rate, the accuracy at the 0.5 threshold, and the AUC with 95% CI using the same procedure as above. In addition, we stratified each outcome by the presence/absence of an epileptic seizure diagnostic suspicion and reported, for each stratum, the outcome prevalence, the balanced accuracy, and the AUC. ROC curves were also computed for each outcome, comparing the full cohort with the epileptic-seizure subgroup and the non-epileptic-seizure subgroup. Between-stratum differences in AUCs were assessed using an independent-samples z-test with DeLong non-parametric variance estimates, with p-values adjusted for multiple comparisons using the Bonferroni correction. For imbalanced outcomes, the DCA threshold range was restricted to clinically plausible values given the observed prevalence. Model interpretability used SHAP to quantify feature contributions to predictions.

### Web-based model deployment

We operationalized all four ML models in a browser-based application hosted on institutional servers (www.emergencyeeg.com). The interface accepts the same admission-time variables used for model training (variable list in Table 1). Upon submission, the application computes class probabilities for (i) abnormal emEEG, (ii) epileptiform emEEG, (iii) emEEG refuting the initial diagnosis, and (iv) emEEG confirming the initial diagnosis, and displays them alongside SHAP-based explanations (patient-level waterfall contribution plots). The platform is free to use, requires no registration, and performs all inference server-side behind HTTPS on institutional infrastructure. To protect privacy and align with ethical approvals for this retrospective study, no user-entered data are stored, logged, or persisted on the server or in the browser beyond the active session; no personally identifiable information is required. The site is intended for research and educational use to support triage reasoning and hypothesis generation rather than to provide stand-alone clinical diagnoses.

**Table 1:** Descriptive statistics of predictors and outcomes in the study population. Values are reported as median and interquartile range (IQR) for continuous variables and absolute and relative frequencies for dichotomous variables

Predictors	Median [IQR] *N* (%)
Age, *years*	70 [29]
Gender, *F*	493 (48.4%)
Initial diagnostic suspicion	
LoC	229 (22.5%)
Altered consciousness	85 (8.3%)
Stroke	131 (12.9%)
TIA	123 (12.1%)
Epileptic seizure	404 (39.7%)
Absence	54 (5.3%)
Status epilepticus	33 (3.2%)
Transient global amnesia	36 (3.5%)
Head trauma	64 (6.3%)
Fall of unknown dynamics	52 (5.1%)
Other	12 (1.2%)
No suspicion	93 (9.1%)
Signs and symptoms, *presence*	
Altered consciousness	267 (26.2%)
LoC	347 (34.1%)
Confusion	353 (34.7%)
Speech disorder	272 (26.7%)
Amnesia	91 (8.9%)
Headache	56 (5.5%)
Weakness	158 (15.5%)
Cranial nerves	63 (6.2%)
Sensory symptoms	63 (6.2%)
Visual symptoms	27 (2.7%)
Hallucinations	8 (0.8%)
Motor manifestations	346 (34.0%)
Morsus	66 (6.5%)
Absence	69 (6.8%)
Regression of symptoms at ED	874 (85.9%)
Anamnestic	
Critical episode witnessed by HCPs	162 (15.9%)
Critical episode witnessed by others	664 (65.2%)
Prior neurosurgery	134 (13.2%)
Known idiopathic epilepsy, *presence*	112 (11.0%)
Known secondary epilepsy, *presence*	168 (16.5%)
Prior brain lesions, *presence*	336 (33.0%)
Fever, *presence*	126 (12.4%)
Sepsis, *presence*	141 (13.9%)
Metabolic alterations, *presence*	83 (8.2%)
Electrolyte alterations, *presence*	154 (15.1%)
Intoxication, *presence*	44 (4.3%)
AED at admission, *presence*	247 (24.3%)
Hypertension, *presence*	435 (42.7%)
Diabetes, *presence*	167 (16.4%)
Dyslipidemia, *presence*	257 (25.2%)
Dysthyroidism, *presence*	121 (11.9%)
Cardiac conditions, *presence*	194 (19.1%)
ED Neurological Conditions	
1 or more neurological conditions	643 (63.2%)
Subdural Hematoma	22 (2.2%)
Tumor	100 (9.8%)
Brain tumor	57 (5.6%)
Neurodegenerative	65 (6.4%)
Trauma	18 (1.7%)
ICH	32 (3.1%)
SAH	15 (1.5%)
Ischemia	106 (10.4%)
Multi-infarct	278 (27.3%)
Other	146 (14.3%)
Neurology referral	234 (23.0%)
Outcomes	
Abnormal emEEG	691 (67.9%)
Epileptiform emEEG	192 (18.9%)
Initial diagnosis ruled out by emEEG	514 (50.5%)
Initial diagnosis confirmed by emEEG	188 (18.5%)

### Exploratory cost scenario analysis

An exploratory cost scenario analysis was performed to provide practical context on resource use under a simulated emEEG workflow prioritization strategy based on model outputs for abnormal and epileptiform outcomes. The analysis was conducted according to standard methodological frameworks for economic evaluation in healthcare [26, 27]. For each model decision threshold, the average cost per patient was computed as:

$$\:{C}_{mean\:}=\:\frac{{C}_{EEG\:}\left(TP+FP\right)+{C}_{penalty\:}FN\:}{N}$$

where TP and FP are the true and false positives, FN are the false negatives; C_EEG_, equal to 150€, is the mean institutional cost of an EEG; C_penalty_ denotes a hypothetical penalty parameter used for sensitivity analysis to represent the consequences of missed or delayed diagnoses. To keep the penalty on the same scale as the modelled resource [28], C_penalty_ was varied from 150€ to 1500€ (i.e., one to ten times the EEG cost).

The relative cost change per patient (RCC/patient) was then calculated as:

$$\:\frac{\mathrm{R}\mathrm{C}\mathrm{C}}{\mathrm{p}\mathrm{a}\mathrm{t}\mathrm{i}\mathrm{e}\mathrm{n}\mathrm{t}}\left(\mathrm{\%}\right)=\:100\cdot\:\frac{{C}_{EEG\:\:}-{C}_{mean\:}\:}{{C}_{EEG\:\:}}$$

## Results

### Cohort Description

**Fig. 1:**
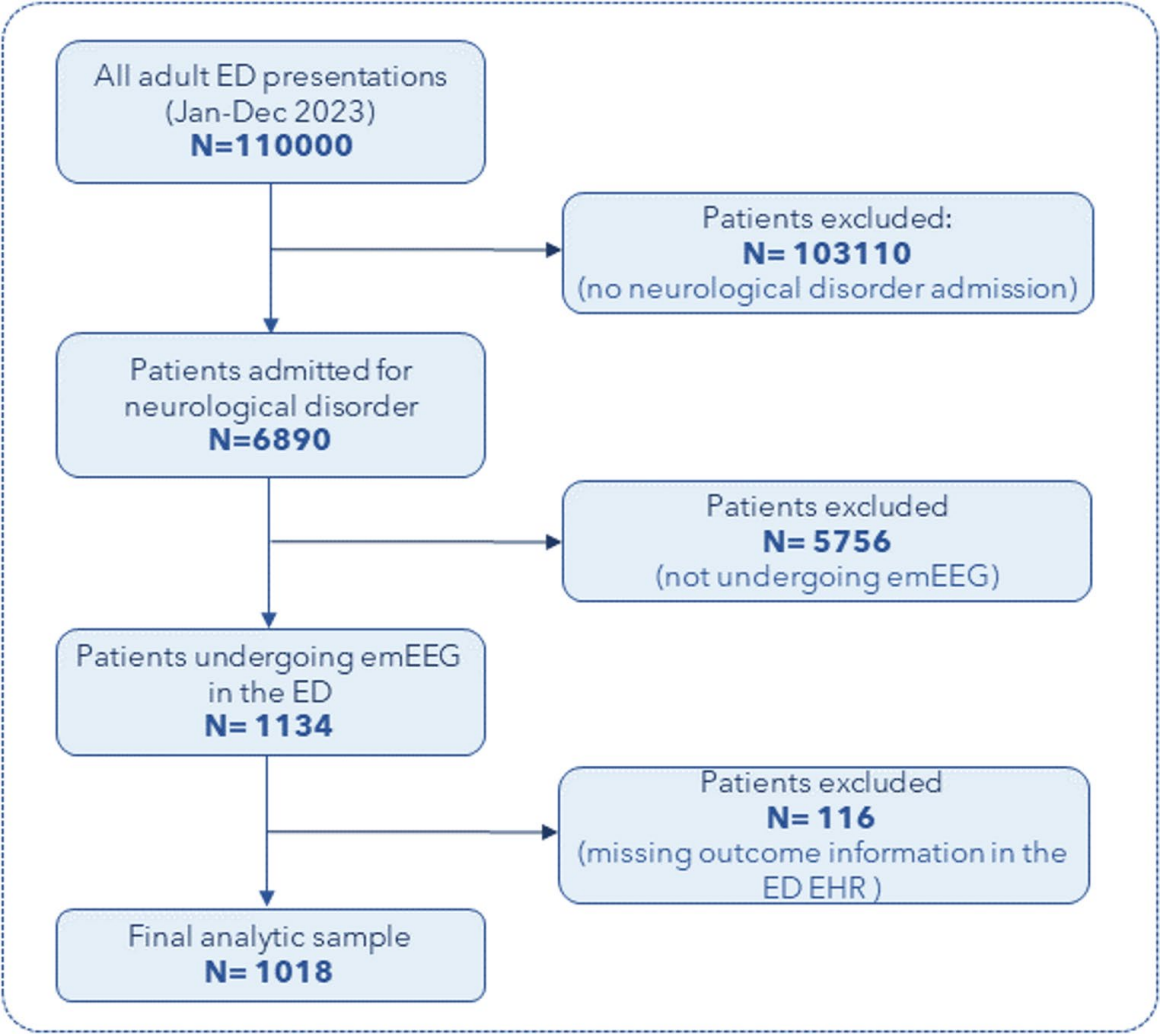
Flow-chart showing the selection process from ED presentations to the final analytic cohort. Legend: emEEG: emergent EEG; ED: Emergency Department; EHR: Electronic Health Record

During the study period, 6890 patients were admitted to the ED for neurological disorders (6.3% of total ED presentations). Among these, 1134 patients underwent emEEG in the ED; outcome data were missing for 116 patients in the electronic health records, resulting in 1018 patients (median age of 70 years [IQR = 29], 48.4% females) included in the analysis (Fig. 1).

The most frequent diagnostic suspicions at ED admission were epileptic seizure (39.7%), loss of consciousness (22.5%), stroke (12.9%), and transient ischemic attack (12.1%). At presentation, confusion (34.7%), loss of consciousness (34.1%), motor manifestations (34.0%), and speech disorder (26.7%) were the most common neurological signs, with symptom regression before EEG occurring in 85.9% of patients. A critical episode (leading to the ED) was witnessed in most cases (81.1%), with 15.9% witnessed by healthcare professionals. Prior brain lesions were present in one-third of the cohort, and 24.3% were on antiepileptic drugs at admission.

Legend: LoC: Loss of Consciousness; TIA: Transient Ischemic Attack; ED: Emergency Department; HCPs: Healthcare Professionals; AED: Antiepileptic Drug; ICH: Intracerebral Hemorrhage; SAH: Subarachnoid Hemorrhage; emEEG: Emergent Electroencephalogram.

Hypertension (42.7%), dyslipidemia (25.2%), and diabetes (16.4%) were the most frequent comorbidities. Overall, 63.2% of patients had at least one neurological condition, most commonly multi-infarct disease (27.3%) or ischemia (10.4%). After the emEEG was performed, abnormal recordings were detected in 67.9% of patients, and epileptiform activity in 18.9%. Diagnostic suspicions with the most abnormal EEG detection were found to be the presence of altered consciousness (94.1%), of status epilepticus (90.9%) and of stroke (79.4%). Coherently, 60.6% and 32.4% of the patients with initial diagnosis of status epilepticus and epileptic seizures (respectively) were found to have an epileptiform EEG. Detailed outcome yields for the subgroups defined by the suspected diagnosis at admission are reported in Supplementary Table S2.

Importantly, emEEG ruled out the initial diagnostic suspicion in 50.5% of cases, while confirming it in 18.5%.

### Prediction of emEEG reporting

**Fig. 2:**
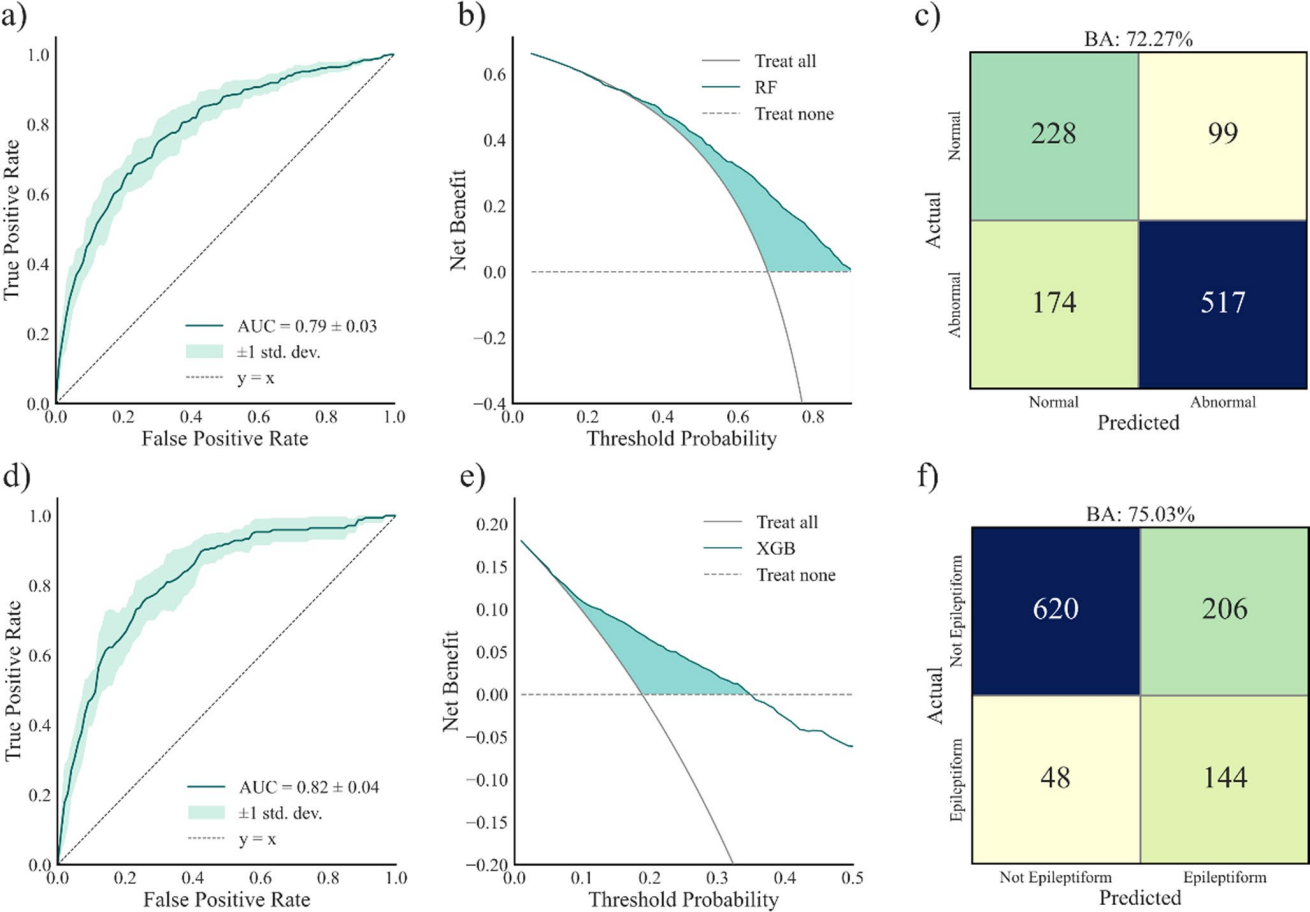
Model performance for abnormal and epileptiform emEEG classification. (**a**) ROC curve for the classification of abnormal emEEG with the Random Forest model. (**b**) Decision curve analysis for abnormal emEEG showing the net benefit of the Random Forest model compared with default strategies. (**c**) Confusion matrix for abnormal emEEG classification at a probability threshold of 50%. (**d**) ROC curve for the classification of epileptiform emEEG with the XGBoost model. (**e**) Decision curve analysis for epileptiform emEEG showing the net benefit of the XGBoost model compared with default strategies. (**f**) Confusion matrix for epileptiform emEEG classification at a probability threshold of 50%.

**Fig. 3:**
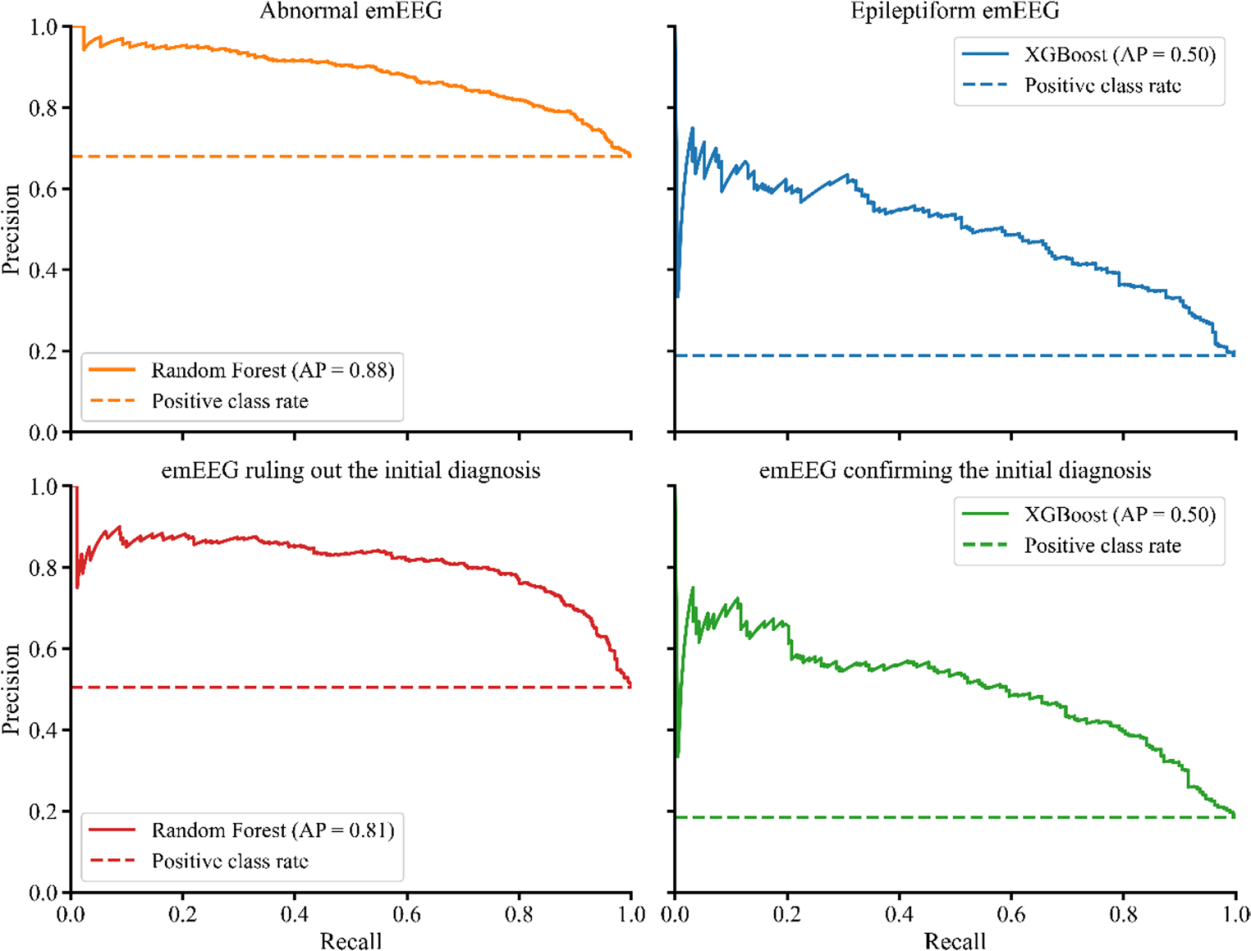
Precision–recall curves for the best-performing models across the four study outcomes (abnormal emEEG, epileptiform emEEG, emEEG ruling out the initial diagnosis, and emEEG confirming the initial diagnosis). Solid lines show precision-recall curves and the dashed horizontal line indicates the outcome prevalence. Average precision (AP) is reported in the legend for each outcome

In the evaluation of abnormal and epileptiform emEEG (Fig. 2), the Random Forest and XGBoost models achieved the best performance in internal validation, respectively. Both models demonstrated a good ability to discriminate between classes, with an AUC of 0.79 (95% CI 0.76–0.82) for abnormal emEEG and 0.82 (95% CI 0.78–0.85) for epileptiform emEEG (Fig. 2A and D, respectively). At a probability threshold of 50%, the balanced accuracy was 72.3%, with sensitivity 74.8% and specificity 69.7% for the classification of abnormal emEEG; for epileptiform emEEG, balanced accuracy was 75.0%, with sensitivity 75.0% and specificity 75.1% (Fig. 2C F, respectively). Precision-recall analysis is reported in Fig. 3. For abnormal emEEG, the F1-score was 0.79, and AP was 0.88; for epileptiform emEEG, the F1-score was 0.53, and AP was 0.50.

The DCA of the model targeting normal/abnormal EEG (Fig. 2B) showed how the model remained above both default strategies (*treat-all* or *treat-none*) across thresholds from 5% to 90%, with a net benefit peaking at 61% at the lowest threshold. When targeting prediction of epileptiform EEGs, a positive net benefit (with a maximum value of 18%) was found up to the threshold of 35%, consistent with the reduced prevalence (18.8%) of the outcome.

**Fig. 4:**
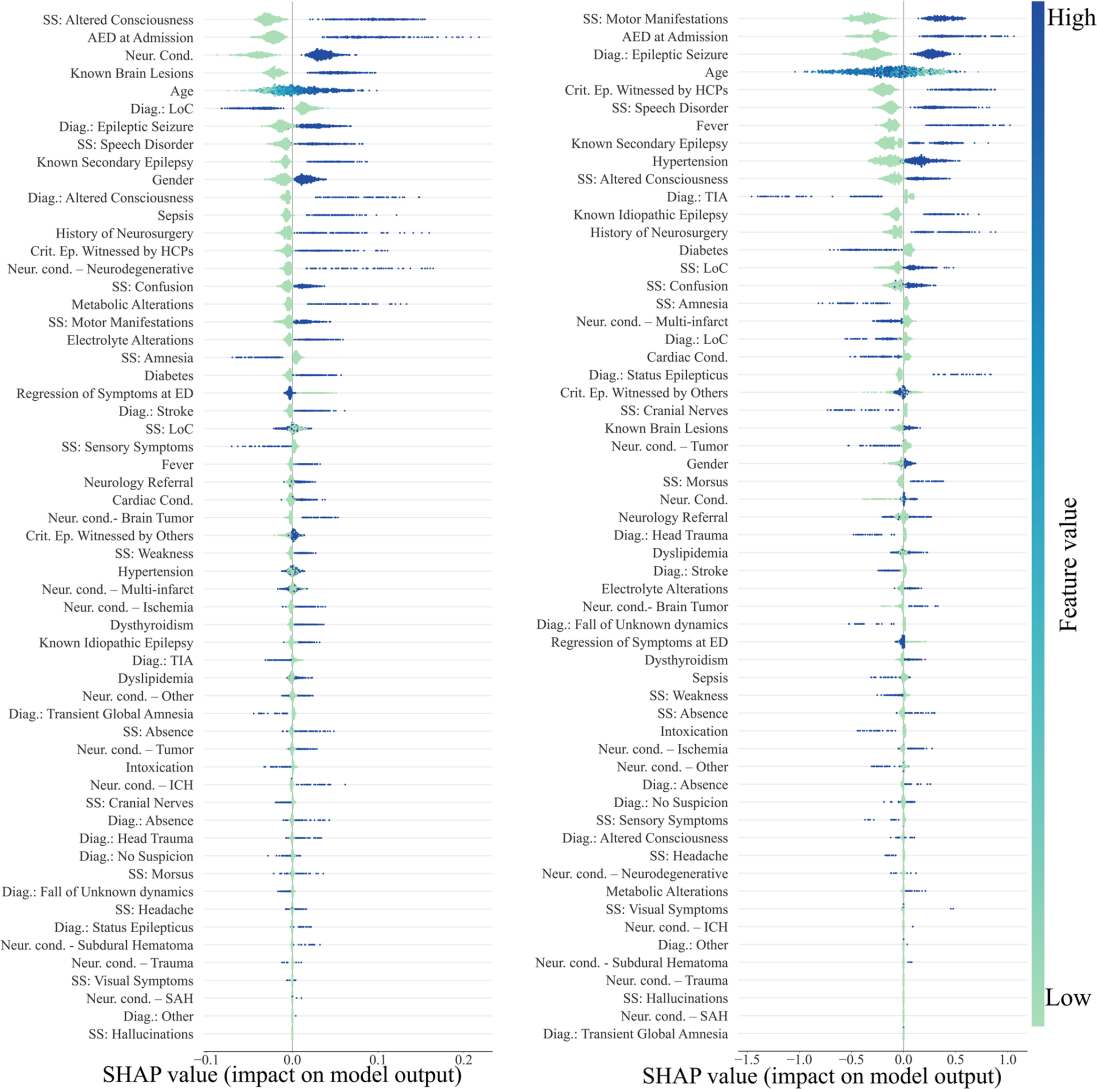
SHAP beeswarm plots with patient-wise feature contributions. Results for the abnormal (left panel) and the epileptiform emEEG classification models were reported. Each row represents a predictor; each point corresponds to one participant. The x-axis shows the SHAP value i.e., the contribution of that predictor to the model output on the model’s native scale. Positive values indicate increased predicted risk/probability of having either an abnormal/epileptiform emEEG; negative values indicate decreased predicted risk/probability of having either an abnormal/epileptiform emEEG. Points are colored by the observed feature value (blue = high, green = low). Features are ordered by global importance (mean absolute SHAP value). Two interpretational examples are provided. First, in the abnormal classification task, SS: Altered Consciousness exhibited an asymmetric effect. While the presence of altered consciousness notably increased the probability of an abnormal classification, its absence almost did not impact the prediction. Second, in the epileptiform classification task, SS: Motor Manifestations displayed a symmetric effect. The presence of motor manifestations consistently increased the probability of an epileptiform classification, whereas their absence decreased this probability to a comparable degree

For abnormal emEEG (Fig. 4, left), the model was mainly driven by features reflecting acute cerebral dysfunction (e.g., altered consciousness at presentation) and chronic neurological vulnerability (such as known brain lesions, prior neurosurgery, and the presence of neurological conditions). The use of antiepileptic drugs at admission further strengthened the likelihood of abnormal findings, pointing to patients already at high neurological risk. Demographic frailty, represented by older age, also consistently pushed predictions toward abnormality. Interestingly, the model leveraged the initial diagnostic suspicion: while LoC tended to favor a prediction of normal EEG, an admission diagnosis of epileptic seizure moved the classification toward abnormality. On the other hand, for epileptiform emEEG (Fig. 4, right), the most influential features were closely aligned with seizure-related clinical activity. Motor manifestations, the presence of antiseizure therapy at admission, and an initial diagnosis of epileptic seizure were the dominant drivers. The reliability of the diagnostic process was reinforced when the critical episode was witnessed by healthcare professionals, which strongly contributed to epileptiform classification. Additional features, such as speech disorder and fever, also pointed in the same direction, though with a more modest effect. Taken together, these findings suggest that the model for abnormal emEEG integrates a broader spectrum of general neurological dysfunction and chronic vulnerability, whereas the model for epileptiform emEEG relies more specifically on acute seizure-related manifestations and clinical context.

### When does emEEG rule-in or rule-out a diagnosis?

**Fig. 5:**
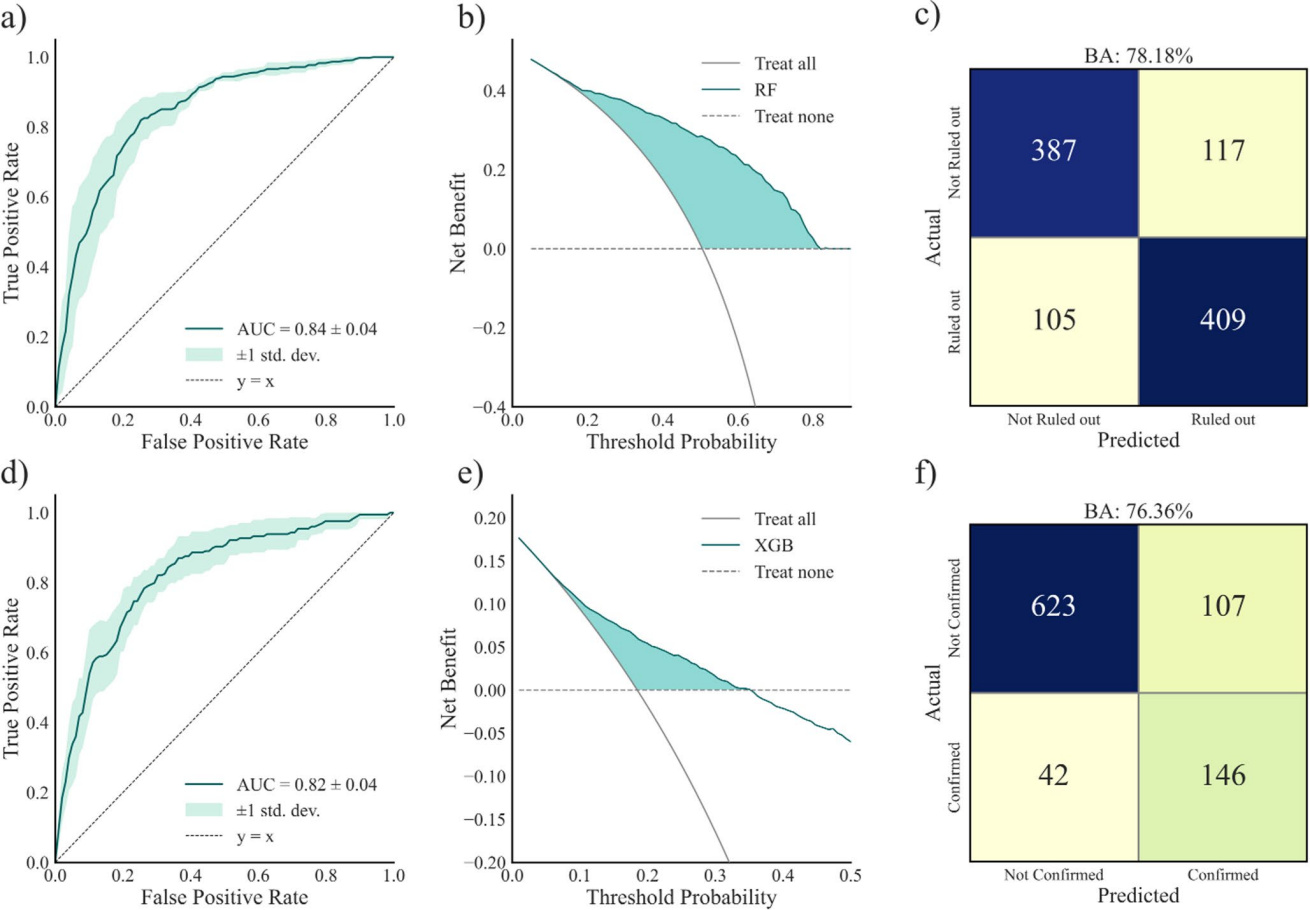
Model performance for emEEG ruling out and confirming the initial diagnosis. (**a**) ROC curve for the classification of emEEG ruling out the initial admission diagnosis with the Random Forest model. (**b**) Decision curve analysis for emEEG ruling out the initial admission diagnosis with comparison against default strategies. (**c**) Confusion matrix for emEEG ruling out the initial admission diagnosis at a probability threshold of 50%. (**d**) ROC curve for the classification of emEEG confirming the initial admission diagnosis with the XGBoost model. (**e**) Decision curve analysis for emEEG confirming the initial admission diagnosis with comparison against default strategies. (**f**) Confusion matrix for emEEG confirming the initial admission diagnosis at a probability threshold of 50%

Legend. AED: AntiEpileptic Drugs; HCP: HealthCare Professionals; ICH: IntraCerebral Haemorrhage; SAH: Subarachnoid Haemorrhage; SHAP: SHapley Additive exPlanations; SS: Signs and Symptoms; TIA: Transient Ischemic Attack.

In the analysis of emEEG ruling out and confirming the initial diagnosis (Fig. 5), the best performance in internal validation was obtained by the Random Forest and XGBoost models, respectively. For the first outcome, the AUC was 0.84 (95% CI 0.81–0.86) (panel a), and, at the threshold of 50%, the balanced accuracy was 78.2%, with sensitivity 79.6% and specificity 76.8% (panel c).

For the second outcome, the AUC was 0.82 (95% CI 0.79–0.85) (panel d) and the balanced accuracy 76.4%, with sensitivity 77.7% and specificity 85.3% (panel f). Precision-recall analysis is reported in Fig. 3. For emEEG ruling out the initial diagnosis, the F1-score was 0.79, and AP was 0.81; for emEEG confirming the initial diagnosis, the F1-score was 0.54, and AP was 0.50. Decision curve analysis (panel b) showed that the Random Forest model yielded a positive net benefit compared with both default strategies (all cases were ruled out or none were ruled out), across thresholds between 5% and 80%, with values of benefit starting at 42% at the lowest threshold. The XGBoost model showed a positive net benefit over both default strategies (all or none cases are confirmed) up to thresholds of 35%, consistent with the lower prevalence of the confirmed outcome (panel e); within this range, net benefit values started at 17%.

Legend. BA: Balanced Accuracy; AUC: Area under the Curve; HCP: HealthCare Professionals; AED: AntiEpileptic Drugs; SS: Signs and Symptoms. RF: Random Forest; XGB: eXtreme Gradient Boosting.

**Fig. 6:**
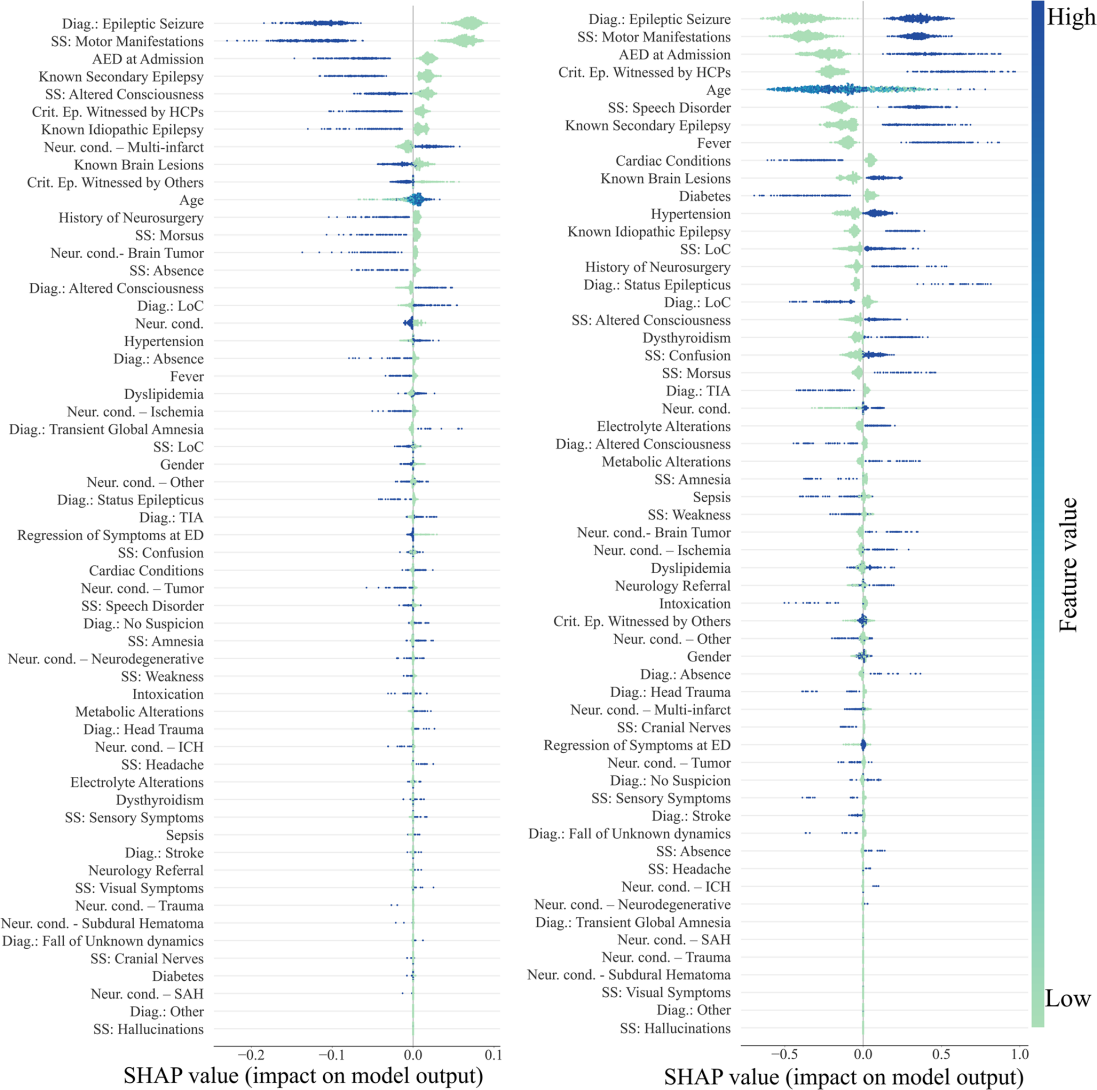
SHAP beeswarm plots with patient-wise feature contributions. Results for the diagnosis rule-out (left panel) and the diagnosis confirmation (right) classification models were reported. Each row represents a predictor; each point corresponds to one participant. The x-axis shows the SHAP value i.e., the contribution of that predictor to the model output on the model’s native scale. Positive values indicate increased predicted risk/probability of the emEEG to rule-out/confirm (left/right panels) the initial diagnosis; negative values indicate decreased predicted risk/probability of the emEEG to not rule-out/not-confirm (left/right panels) the initial diagnosis. Points are colored by the observed feature value (blue = high, green = low). Features are ordered by global importance (mean absolute SHAP value). Legend. AED: AntiEpileptic Drugs; HCP: HealthCare Professionals; ICH: IntraCerebral Haemorrhage; SAH: Subarachnoid Haemorrhage; SHAP: SHapley Additive exPlanations; SS: Signs and Symptoms; TIA: Transient Ischemic Attack

Beyond this shared core, the models diverge in context, and witness reliability separates the two predictions (Fig. 6): events witnessed by healthcare professionals support confirmation and tend to oppose rule-out. Motor manifestations, AED use at admission, and known secondary epilepsy show the same directional pattern; in the rule-out model, episodes witnessed by others also appear, but as a weaker signal. Seizure-specific signs such as morsus (tongue bite) and “absence” episodes counter rule-out, coherently opposing the rule-out decision. In the confirm model, speech disorder features prominently and favors confirmation as a feature aligned with an epileptic phenotype.

Structural and cumulative vulnerability further characterizes the rule-out profile. The rule-out model gives weight to multi-infarct disease and older age, which together shift predictions toward rule-out and away from a seizure-driven interpretation of the index event. By contrast, in the confirm model known secondary epilepsy favors confirmation, and known idiopathic epilepsy, known brain lesions, and prior neurosurgery similarly support confirmation, consistent with a pre-existing neurological vulnerability. Finally, systemic comorbidity is more visible in the confirm model as negative contributors: cardiac conditions and diabetes show the strongest negative impact, and their presence reduces the likelihood of confirmation, coherently pointing to non‑epileptic or multifactorial etiologies. Their relative absence among the top rule‑out drivers suggests that, for refutation, structural/neurologic burden and non‑specific cerebral dysfunction dominate over general medical comorbidity.

### Error Analysis

**Fig. 7:**
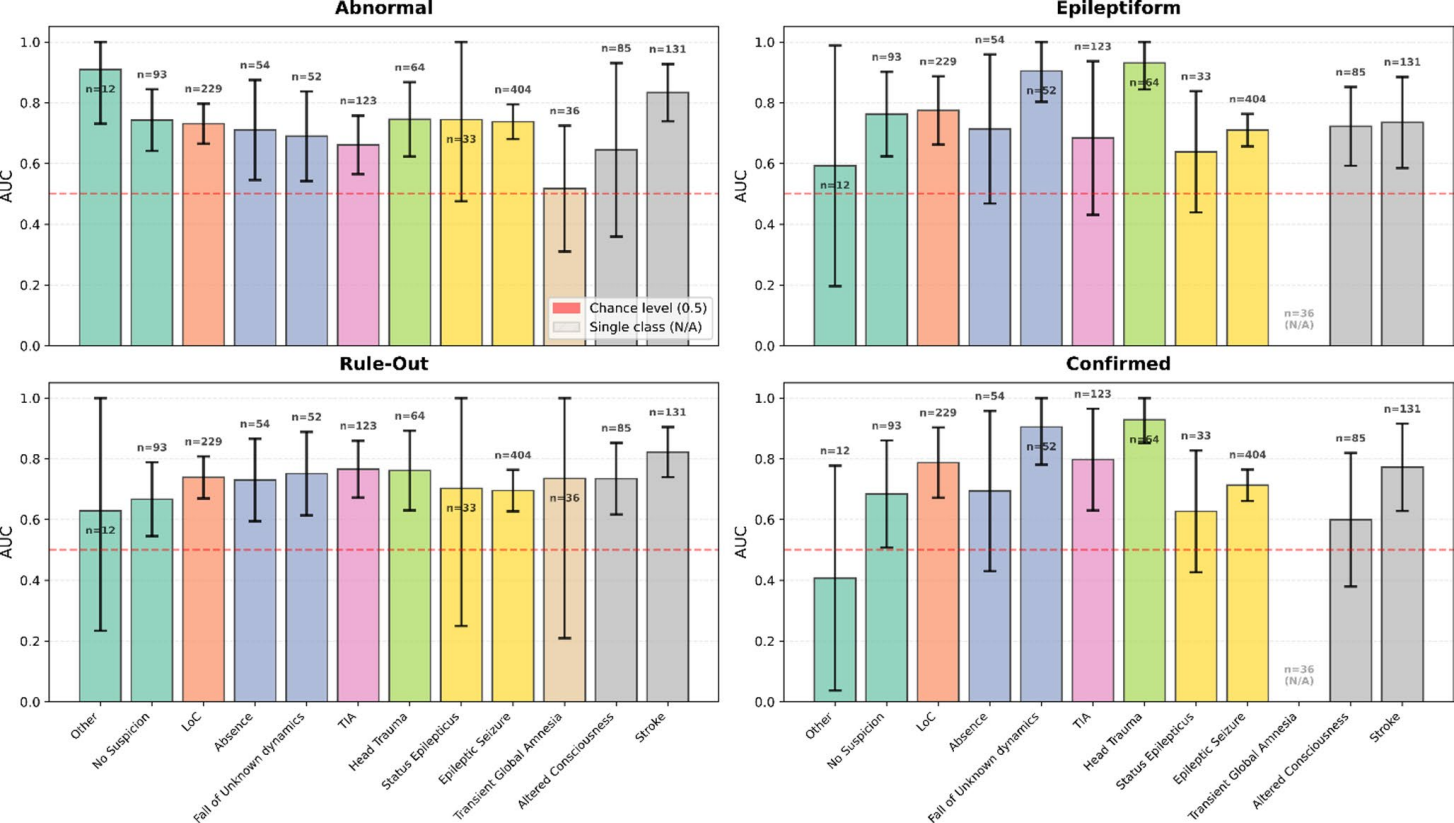
AUC with 95% confidence intervals by admission diagnostic-suspicion subgroup for the four different outcomes. Within each panel, vertical bars depict the test-set AUC for each subgroup, with black error bars showing 95% CIs and a horizontal dashed red line marking chance level (AUC = 0.5) for binary outcomes. Numbers above the bars indicate the subgroup sample size of the specific diagnostic subgroup. Bars with hatched gray fill denote subgroups where AUC was not estimable (single observed class)

Evaluation metrics (AUCs) were grouped using the twelve diagnostic suspicions at admission, with strata ranging from 12 to 404 patients (Fig. 7). Regarding the assessment of whether models correctly predicted EEG abnormality, AUCs were consistently above chance and generally clustered in the 0.7–0.8 range, with narrower CIs in the largest strata (e.g., LoC, Epileptic Seizure, Stroke). Visibly, only for patients diagnosed with Transient Global Amnesia, the models’ prediction capability had an accuracy of 66.7% (AUC of 0.52, 95% CI 0.31–0.72), close to chance level.

Concerning epileptiform EEG prediction, eleven of twelve subgroups were evaluable (patients with transient global amnesia were never found to be epileptiform). Discrimination was highest for patients after a trauma (AUC of 0.93, 95% CI 0.84-1.00, *n* = 64) or after a fall of unknown dynamics (AUC of 0.91, 95% CI 0.80-1.00; *n* = 52). Large strata showed stable performance, e.g., patients with a loss of consciousness (AUC of 0.78, 95% CI 0.66–0.89; *n* = 229), or with epileptic seizures (AUC of 0.71, 95% CI 0.66–0.76; *n* = 404). The sole category that reached an AUC below 60% was the one termed Other (including 12 patients with a non-missing suspicion but not included in the proposed categories).

**Fig. 8:**
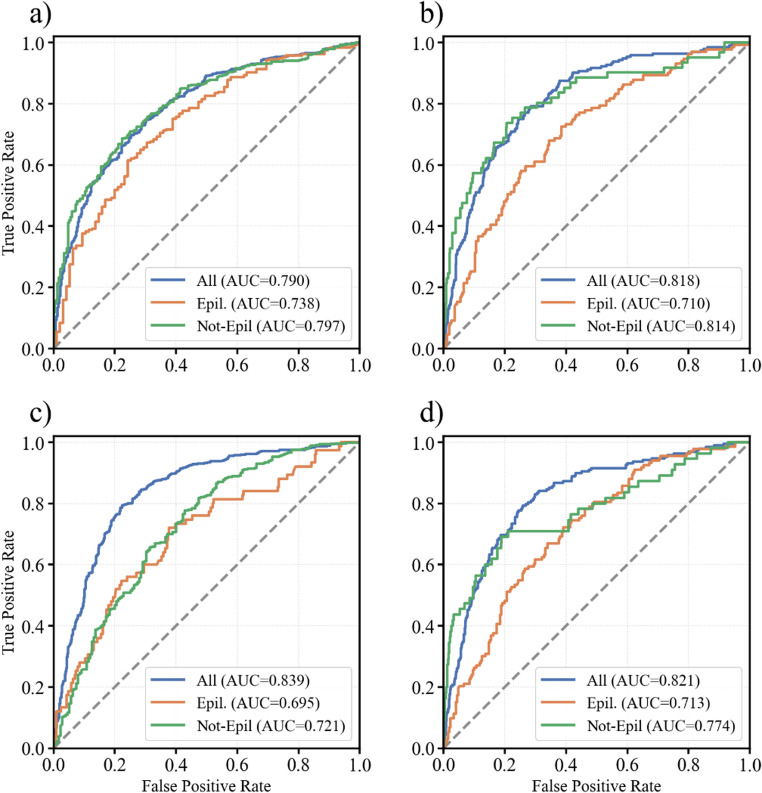
ROC curves for the four outcomes stratified by initial diagnostic suspicion of epileptic seizure and for the full cohort. Panels: (**a**) abnormal emEEG, (**b**) epileptiform emEEG, (**c**) emEEG ruling out the initial diagnosis, (**d**) emEEG confirming the initial diagnosis

When stratifying by initial diagnostic suspicion of epileptic seizure versus other admission diagnostic suspicions, performance remained above chance and showed a consistent direction of change across outcomes (Fig. 8; Table 2). Discrimination was generally lower in the epileptic-seizure stratum than in the non-epileptic-seizure stratum, with the largest attenuation observed for the rule-out outcome, in parallel with the marked prevalence shift between strata. Between-stratum AUC comparisons did not reach statistical significance for any outcome (Table 2).

**Table 2:** Performance summary for each outcome, stratified by initial diagnostic suspicion of epileptic seizure. For each outcome and group, the table reports prevalence, test balanced accuracy, and AUC (95% CI; DeLong non-parametric method). Additionally, the table presents the effect size and Bonferroni-adjusted p-values from an independent-samples z-test

Outcome	Group	Balanced Accuracy	Prevalence	AUC (95% CI)	ΔAUC	*p*-value
Abnormal	Epil. (*N* = 404)	0.67	0.76	0.74 (0.68–0.80)	-0.059	0.333
	Not-Epil. (*N* = 614)	0.73	0.62	0.80 (0.76–0.83)		
Epileptiform	Epil. (*N* = 404)	0.63	0.32	0.71 (0.66–0.77)	-0.104	0.061
	Not-Epil. (*N* = 614)	0.71	0.10	0.81 (0.75–0.88)		
emEEG ruling out	Epil. (*N* = 404)	0.55	0.19	0.70 (0.62–0.76)	-0.026	1.000
	Not-Epil. (*N* = 614)	0.65	0.71	0.72 (0.68–0.77)		
emEEG confirming	Epil. (*N* = 404)	0.64	0.33	0.71 (0.66–0.77)	-0.062	0.768
	Not-Epil. (*N* = 614)	0.70	0.09	0.77 (0.70–0.85)		

### Downstream Applications

**Fig. 9:**
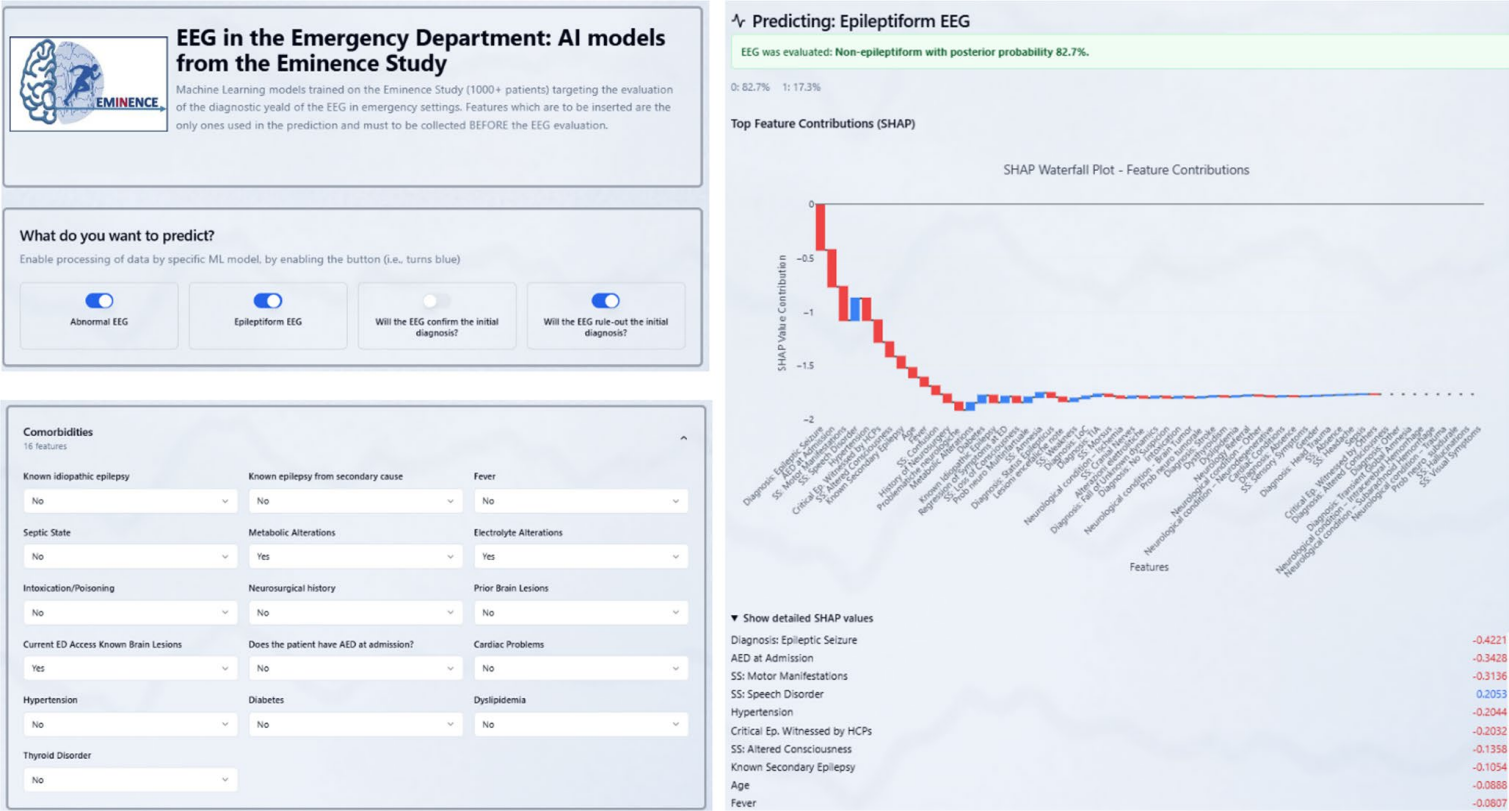
Public web interface for ML model deployment. (a) Input panel with possibility of selecting prediction tasks. (b) Input panel for the admission variables (demographics, initial diagnostic labels, signs/symptoms, anamnestic and comorbidity fields, medication, and ED neuroimaging flags) required for model prediction. (c) Output panel providing predicted probabilities for each task combined with SHAP-based explanations by means of a patient-level waterfall plot decomposing the individual prediction

Furthermore, a public web tool (www.emergencyeeg.com) was released to enable external users to reproduce our predictions from admission-time inputs and to visualize feature attributions for individual cases. The interface (Fig. 9) presents three core components: (i) a descriptive part enabling which task(s) to perform, (ii) a structured form mirroring the study variables, and (iii) an output panel with task-specific probabilities and SHAP explanations.

Per-patient plots show the direction and magnitude of each feature’s contribution to the predicted outcome, making also explicit reference to the numerical SHAP value for specific feature-patient combination.

### Exploratory cost scenario results

**Fig. 10:**
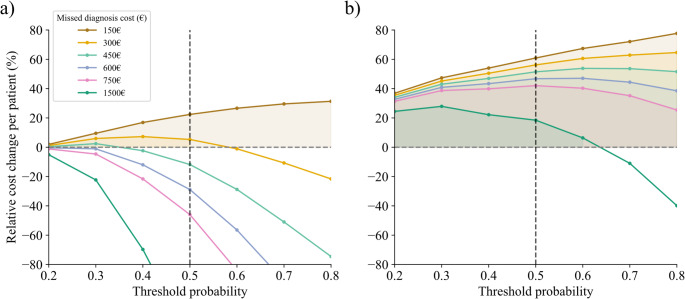
Relative cost change per patient across different model decision thresholds and missed diagnosis penalties for abnormal (panel **a**) and epileptiform (panel **b**) emEEG classifications

Under the exploratory cost scenario analysis, a simulated workflow in which emEEGs were prioritized only when the model estimated an abnormal result produced positive RCC/patient values only under low assumed penalty costs (Fig. 10, panel a). The maximum simulated saving reached approximately 20% at a decision threshold of 0.5 and a penalty cost of 150 €, while higher penalty scenarios progressively reduced the advantage, leading to losses when the cost of a missed diagnosis exceeded 300 €. By contrast, for epileptiform outcomes, the simulation showed positive RCC/patient values across most tested thresholds and penalty assumptions (Fig. 10, panel b). At the reference threshold of 0.5, RCC/patient values ranged between 18% and 61%, and savings persisted even at the highest simulated penalty level (1500 €, i.e., 10 times the EEG cost).

## Discussion

This study demonstrated that admission-time data were able to predict (a) abnormal/epileptiform emEEG and (b) whether emEEG would confirm or refute the ED diagnosis. Discrimination was good (AUC ~ 0.79–0.84; balanced accuracy ~ 72–78%). Decision-curve analysis showed that, across practical and prevalence-weighted cut-offs, using the models yields more correct clinical actions than doing emEEG for everyone or for no one. Discrimination was good for identifying abnormal recordings (balanced accuracy 72%, AUC ≈ 0.79) and epileptiform activity (balanced accuracy 75%, AUC ≈ 0.82). The models were also informative regarding the diagnostic contribution of emEEG in relation to the admission diagnosis, predicting whether the EEG findings ruled out (balanced accuracy 78%, AUC ≈ 0.84) or confirmed (balanced accuracy 76%, AUC ≈ 0.82) the initial suspicion. Given the lower outcome prevalence of epileptiform emEEG and diagnosis confirmation, we also evaluated precision-recall performance. AP was ~ 0.50, compared with positive class rates of ~ 0.19, and F1-scores were 0.53–0.54. Error analyses showed that performance varied across initial diagnostic-suspicion subgroups. These subgroups were unevenly represented in our cohort, but discrimination remained consistently above chance. When stratifying patients by epileptic seizure versus non-epileptic seizure admission suspicion, discrimination was not higher in the epileptic-seizure stratum, indicating that cohort-level performance was not inflated by the predominance of this subgroup. Between-stratum AUC comparisons did not reach statistical significance and were consistent with this overall pattern.

These results suggest potential utility of these models as practical aids to support triage decisions, complementing but not replacing clinical judgment. Model interpretability clarifies when emEEG is most likely to be informative and how it informs clinical reasoning. For abnormal versus normal emEEG, the model focuses on giving importance to acute cerebral dysfunction, especially altered consciousness at presentation; it also considers chronic neurological vulnerability (known brain lesions, prior neurosurgery, pre-existing neurological conditions), together with AED use at admission and older age. These patterns align with our previous analysis in the same cohort and with ED series reporting higher emEEG yield in altered mental status and status epilepticus, whereas normal recordings are more common in global amnesia, minor head trauma, and falls of unknown origin [1, 4, 6]. The present results add directional nuance (interpretational example Fig. 4), offering more specific patient-wise diagnostic information. For example, loss of consciousness as a stand-alone label pushed predictions toward a normal recording, while its presence had a notably smaller impact on the likelihood of the ML model to classify the EEG as abnormal. In addition, speech disorder and fever emerged as positive contributors to an epileptiform recording; this pattern suggests a probable connection with seizure propensity and with encephalopathic states in which seizures are common.

The SHAP analysis for diagnostic refutation versus confirmation delineates complementary clinical profiles and supports a pragmatic use of emEEG. Three features, i.e., admission diagnosis of epileptic seizure, motor manifestations, and AED at admission, were the most influential in both models but acted in opposite directions: they increased the probability of confirmation and decreased the probability of refutation. The symmetric bidirectional contribution of these features strengthens the clinical plausibility of the findings and mirrors the pre-test diagnostic reasoning typically applied in seizure triage, thereby supporting the pragmatic utility of the model. In particular, when classic seizure cues are present (ED seizure diagnosis, motor signs, AED given), it predicts emEEG will confirm; when they are weak or absent, it predicts emEEG will refute. This mirror‑image effect across the two models supports the clinical credibility and everyday usability of the approach.

However, beyond this shared triad, the models diverged in clinically coherent ways. Confirmation was amplified by reliability factors such as events witnessed by healthcare professionals and by features aligned with an epileptic phenotype (for example, speech disorder). By contrast, rule-out was driven by a broader profile compatible with non-specific presentations and vascular or structural burden, including admission diagnostic labels such as altered consciousness or loss of consciousness, older age, and multi-infarct disease, whereas seizure-specific signs such as morsus and “absence” episodes opposed rule-out. Systemic comorbidity, particularly cardiac disease and diabetes, emerged in the confirmation model as negative contributors, reducing the likelihood that emEEG would confirm the initial diagnostic category and suggesting greater diagnostic complexity.

To this extent, the subgroup-specific results reinforce the effect shown by the signs and symptoms at presentation: seizure-centric contexts (e.g., head trauma with motor phenomena, or clear LoC) favor epileptiform/confirmation discrimination, whereas structural or nonspecific encephalopathic contexts favor rule-out. The width of the confidence intervals tracks subgroup size and case heterogeneity, providing an uncertainty signal that is directly actionable at the bedside. Reporting CIs alongside point estimates therefore helps calibrate clinicians’ trust in the predictions for a given diagnostic cohort in a personalized manner.

In the ED, where clinicians must provide a diagnostic assessment of each patient even before full testing, the model’s patterns suggest a pragmatic rule: when seizure features cluster and the index event is clearly witnessed, emEEG is likely to confirm; when presentations are nonspecific, confounded by systemic illness, or occur on a background of structural brain disease, emEEG is more likely to redirect. This reconciles the frequent observation that emEEG contributes to decision-making even when it is normal or non-epileptiform [3, 11, 15–17].

Beyond diagnostic accuracy, an exploratory cost scenario analysis was performed to provide practical context on resource use under a simulated model-guided emEEG prioritization workflow. The model predicting emEEG abnormalities showed only modest benefit, with savings limited to scenarios in which the assumed penalty for a missed diagnosis was relatively low (approximately up to 1–2 times the EEG cost), consistent with the heterogeneous and often not only epileptic-related nature of abnormal emEEG findings. In contrast, the model predicting epileptiform activity yielded a more favorable economic profile, with savings remaining evident across the full range of penalty values explored, including scenarios in which the missed-diagnosis penalty was set at up to ten times the EEG cost. These findings suggest that model outputs may support workflow prioritization in clinically stable or non-critical presentations, where resource allocation is a relevant consideration. However, in highly acute or high-risk presentations, emEEG should remain the default approach given the time-sensitive implications of diagnostic and treatment decisions. This scenario analysis has important limitations: the consequences of missed or delayed diagnoses were represented using a simplified penalty parameter rather than empirically estimated downstream costs; the framework does not capture rare but high-impact clinical trajectories, diagnosis-specific consequences, or patient-level heterogeneity; and the resulting cost estimates are not directly transferable across institutions or healthcare systems, where both resource costs and the clinical consequences of delay may differ substantially.

Taken together, the results complement and extend prior literature on emergency neurodiagnostics and align with contemporary guidance that emphasizes timely EEG for suspected seizures/NCSE while recognizing variability in yield across indications [5, 8–10, 13]. They also fit the broader trajectory of AI in acute care as a decision-support adjunct: interpretable models that integrate clinician-curated ED variables and neuroimaging can surface transparent reasons for triage choices and reduce unwarranted testing, in line with views that AI should augment rather than replace clinician judgment [23–25].

Strengths include a large cohort with structured access to emEEG, rigorous nested cross-validation, and decision-curve analysis, together with explanations that accord with pathophysiology and prior empirical observations.

Limitations include the retrospective single-center design, the presence of outcome imbalance in the epileptiform and confirmation classes, and the non-uniform distribution of admission diagnostic suspicions in the cohort, all of which may affect cohort-level performance estimates and limit generalizability.The non-uniformity of admission suspicions likely reflects the clinical pathway through which emEEG was requested, as the test was ordered according to physicians’ judgment rather than systematically applied to all ED presentations. Consequently, seizure-related presentations are likely overrepresented compared with the broader ED population, and this selection process should be considered when interpreting cohort-level performance summaries and generalizability. In addition, cohort composition (Table 1) constrains external validity and fairness beyond similar adult ED populations; the model is not intended for pediatric ED patients or for settings without timely emEEG acquisition and expert interpretation (e.g., no 24-hour EEG coverage), and it may not generalize to institutions with substantially different referral pathways. Moreover, because the study was conducted at a single center, local clinical workflows and institutional practices-including neurology referral thresholds, emEEG ordering criteria and urgency, and operational resource constraints-may shape both the case-mix undergoing emEEG and the relationship between admission predictors and emEEG-defined outcomes. As a result, these factors may potentially affect performance when the models are applied in other clinical settings.

Future research should prospectively evaluate these models in multicenter ED cohorts that are geographically and temporally independent, explicitly reflecting differences in experimental set-up, referral workflows, and admission diagnostic distributions; quantify effects on time to diagnosis, treatment changes, and avoidance of unnecessary antiseizure therapy in rule-out profiles; and assess cost-effectiveness. External validation, calibration monitoring, and fairness analyses are warranted.

To this extent, the public deployment of the models may enable clinicians and researchers to explore model behavior using locally relevant admission profiles and to visualize why predictions are made, thereby supporting triage reasoning and external validation of the solution. Methodologically, immediate access to SHAP explanations may enhance interpretability and facilitate external scrutiny, which are relevant for decision-support tools intended to complement, rather than replace, clinical judgment. Importantly, the platform does not store any user inputs, adhering to EU privacy requirements.

In conclusion, interpretable ML models using only admission data can identify patients with a higher yield for abnormal and epileptiform emEEG and can anticipate whether the test will confirm or refute the initial diagnostic suspicion. The explanatory patterns map closely onto clinical reasoning, with seizure-centric profiles aligned with confirmation and systemic or structural profiles aligned with refutation. This may facilitate emEEG triage and reinforce ML as decision support in emergency neurology rather than a replacement for clinician judgment.

### Data Availability

The datasets are not publicly available due to privacy and ethical restrictions or institutional policies, but are available from the corresponding author upon reasonable request.

### Code Availability

Code used to develop and train the models, and to reproduce figures in the manuscript will be made publicly available upon acceptance. The models and explanatory tools developed in this study are available on a freely accessible website (www.emergencyeeg.com).

## Supplementary Information

Below is the link to the electronic supplementary material.

Supplementary Material 1 (DOCX 16.8 KB)

Supplementary Material 2 (DOCX 16.4 KB)

## Data Availability

The datasets are not publicly available due to privacy and ethical restrictions or institutional policies, but are available from the corresponding author upon reasonable request.
