# Supplementary material for: Interpretable Machine Learning to Anticipate the Diagnostic Yield of EEG in the Emergency department. The EMINENCE study
Source: J Med Syst. 2026 May 1;50(1):67. doi: 10.1007/s10916-026-02397-y (PMC13132943; doi:10.1007/s10916-026-02397-y)
Supplement: Supplementary file 1 — Supplementary Material 1 (DOCX 16.8 KB) [file 10916_2026_2397_MOESM1_ESM.docx]

**Interpretable Machine Learning to Anticipate the Diagnostic Yield of EEG in the Emergency department. The EMINENCE study**

Maenia Scarpino^1^, Ester Marra^2,*^, Piergiuseppe Liuzzi^2^, Benedetta Piccardi^3^, Peiman Nazerian^4^, Ilaria Sgrilli^1^, Andrea Mannini^2^, Andrea Nencioni^4, †^, Antonello Grippo^1,†^

^†^Dr. Antonello Grippo and Dr. Andrea Nencioni contributed equally to the manuscript

^1^Neurophysiopathology Unit, Careggi University Hospital, Florence, Italy

^2^IRCCS Don Carlo Gnocchi ONLUS, Florence, Italy

^3^Stroke Unit, Careggi University Hospital, Florence, Italy

^4^Emergency Department, Careggi University Hospital, Florence, Italy

*Correspondence to: Ester Marra, [emarra@dongnocchi.it](mailto:emarra@dongnocchi.it), +39 3394494745, IRCCS Fondazione Don Carlo Gnocchi ONLUS, Firenze, Via di Scandicci 269, Fi, IT.

**Supplementary Table S1.** Hyperparameter optimization ranges explored for Random Forest and XGBoost models within the inner loops of nested cross-validation.

| **Hyperparameter** | **Description** | **Optimization range** |
| --- | --- | --- |
| **Random Forest** | | |
| ccp_alpha | Complexity parameter for pruning | 0.0-0.02 |
| min_samples_split | Minimum samples to split a node | 10-50 |
| min_samples_leaf | Minimum samples in a leaf | 5-20 |
| max_depth | Maximum depth of the tree | 2-8 |
| n_estimators | Number of trees | 1-100 |
| **XGBoost** | | |
| learning_rate | Learning rate | 0.005-0.1 |
| max_depth | Maximum depth of each tree | 2-8 |
| min_child_weight | Minimum weight required in a child node | 5-30 |
| colsample_bytree | Fraction of features used per tree | 0.5-0.9 |
| n_estimators | Number of trees | 1-100 |
| gamma | Minimum loss reduction for a split | 0-5 |
| scale_pos_weight | Balance factor for positive class weights | 4-6.5 |
