# Supplementary material for: Interpretable Machine Learning to Anticipate the Diagnostic Yield of EEG in the Emergency department. The EMINENCE study
Source: J Med Syst. 2026 May 1;50(1):67. doi: 10.1007/s10916-026-02397-y (PMC13132943; doi:10.1007/s10916-026-02397-y)
Supplement: Supplementary file 2 — Supplementary Material 2 (DOCX 16.4 KB) [file 10916_2026_2397_MOESM2_ESM.docx]

**Interpretable Machine Learning to Anticipate the Diagnostic Yield of EEG in the Emergency department. The EMINENCE study**

Maenia Scarpino^1^, Ester Marra^2,*^, Piergiuseppe Liuzzi^2^, Benedetta Piccardi^3^, Peiman Nazerian^4^, Ilaria Sgrilli^1^, Andrea Mannini^2^, Andrea Nencioni^4, †^, Antonello Grippo^1,†^

^†^Dr. Antonello Grippo and Dr. Andrea Nencioni contributed equally to the manuscript

^1^Neurophysiopathology Unit, Careggi University Hospital, Florence, Italy

^2^IRCCS Don Carlo Gnocchi ONLUS, Florence, Italy

^3^Stroke Unit, Careggi University Hospital, Florence, Italy

^4^Emergency Department, Careggi University Hospital, Florence, Italy

*Correspondence to: Ester Marra, [emarra@dongnocchi.it](mailto:emarra@dongnocchi.it), +39 3394494745, IRCCS Fondazione Don Carlo Gnocchi ONLUS, Firenze, Via di Scandicci 269, Fi, IT.

**Supplementary Table S2.** Outcome yields by admission diagnostic-suspicion subgroup. For each subgroup, the table reports the number and percentage of positive outcomes for Abnormal, Epileptiform, Rule-Out, and Confirmed tasks.

| Diagnosis | Abnormal | Epileptiform | Rule-Out | Confirmed |
| --- | --- | --- | --- | --- |
| LoC | 109 (47.6%) | 24 (10.5%) | 145 (63.3%) | 22 (9.6%) |
| Absence | 38 (70.4%) | 10 (18.5%) | 21 (38.9%) | 9 (16.7%) |
| Fall of Unknown dynamics | 31 (59.6%) | 3 (5.8%) | 33 (63.5%) | 3 (5.8%) |
| TIA | 70 (56.9%) | 6 (4.9%) | 84 (68.3%) | 9 (7.3%) |
| Head Trauma | 43 (67.2%) | 6 (9.4%) | 42 (65.6%) | 6 (9.4%) |
| Status Epilepticus | 30 (90.9%) | 20 (60.6%) | 4 (12.1%) | 20 (60.6%) |
| Epileptic Seizure | 309 (76.5%) | 131 (32.4%) | 75 (18.6%) | 133 (32.9%) |
| Transient Global Amnesia | 12 (33.3%) | 0 (0.0%) | 34 (94.4%) | 0 (0.0%) |
| Altered Consciousness | 80 (94.1%) | 14 (16.5%) | 57 (67.1%) | 9 (10.6%) |
| Stroke | 104 (79.4%) | 17 (13.0%) | 78 (59.5%) | 17 (13.0%) |
| Other | 11 (91.7%) | 3 (25.0%) | 7 (58.3%) | 3 (25.0%) |
| No Suspicion | 60 (64.5%) | 11 (11.8%) | 68 (73.1%) | 11 (11.8%) |

**Legend.** TIA: Transient Ischemic Attack; LoC: Loss of Consciousness
